# Characteristics of Clinical Shiga Toxin-Producing *Escherichia coli* Isolated from British Columbia

**DOI:** 10.1155/2013/878956

**Published:** 2013-10-02

**Authors:** Kevin J. Allen, Chad R. Laing, Ana Cancarevic, Yongxiang Zhang, Lili R. Mesak, Hai Xu, Ana Paccagnella, Victor P. J. Gannon, Linda Hoang

**Affiliations:** ^1^Food, Nutrition and Health Program, Faculty of Land and Food Systems, University of British Columbia, 218-2205 East Mall, Vancouver, BC, Canada V6T 1Z4; ^2^Laboratory for Foodborne Zoonoses, Public Health Agency of Canada, 225089 Township Road 9-1 (Box 640), Lethbridge, AB, Canada T1J 3Z4; ^3^The State Key Laboratory of Microbial Technology, School of Life Science, Shandong University, Jinan 250100, Shandong, China; ^4^BCCDC Public Health and Reference Microbiology Laboratory, PHSA, 655 West 12th Ave, Vancouver, BC, Canada V5Z 4R4; ^5^Department of Pathology and Laboratory Medicine, University of British Columbia, 2211 Wesbrook Mall, Vancouver, BC, Canada V6T 2B5

## Abstract

Shiga toxin-producing *Escherichia coli* (STEC) are significant public health threats. Although STEC O157 are recognized foodborne pathogens, non-O157 STEC are also important causes of human disease. We characterized 10 O157:H7 and 15 non-O157 clinical STEC derived from British Columbia (BC). *Eae, hlyA,* and *stx* were more frequently observed in STEC O157, and 80 and 100% of isolates possessed *stx*_1_ and *stx*_2_, respectively. In contrast, *stx*_1_ and *stx*_2_ occurred in 80 and 40% of non-O157 STEC, respectively. Comparative genomic fingerprinting (CGF) revealed three distinct clusters (C). STEC O157 was identified as lineage I (LI; LSPA-6 111111) and clustered as a single group (C1). The *cdi* gene previously observed only in LII was seen in two LI O157 isolates. CGF C2 strains consisted of diverse non-O157 STEC while C3 included only O103:H25, O118, and O165 serogroup isolates. With the exception of O121 and O165 isolates which were similar in virulence gene complement to STEC O157, C1 O157 STEC produced more Stx2 than non-O157 STEC. Antimicrobial resistance (AMR) screening revealed resistance or reduced sensitivity in all strains, with higher levels occurring in non-O157 STEC. One STEC O157 isolate possessed a mobile *bla*_CMY-2_ gene transferrable across genre via conjugation.

## 1. Introduction

*Escherichia coli* are Gram negative, facultative anaerobic bacteria found in mammalian gastrointestinal tracts. *Escherichia coli* possessing Shiga toxin genes (*stx*) (i.e., Shiga toxin-producing *E. coli* [STEC]) pose serious health risks through consumption of contaminated food [1–4]. Classical enterohemorrhagic *E. coli* encode *stx*, plasmid pO157 (*hlyA*), and the locus of enterocyte effacement (LEE); however, LEE negative strains may also cause severe disease [3], the most notable example being *E. coli* O104:H4 [5].

 In 2003, Karmali et al. [6] grouped STEC into seropathotypes based on serogroup occurrence in human disease, the capacity to cause outbreaks, and the association with hemolytic uremic syndrome (HUS). STEC O157:H7 and O157:NM were identified as the most significant public health risk (seropathotype A), whereas non-O157 STEC are progressively of lower risk from groups B to E. Recent estimates suggest that STEC O157 causes 50 to 70% of human infections, meaning that 30 to 50% are caused by non-O157 STEC [7–10]. A US study examining high-risk non-O157 STEC in beef revealed that 1006 of 4133 samples were positive for STEC by PCR, though only 10 isolates possessed virulence gene combinations of known pathogens [11]. Outside North America, non-O157 STEC are well-recognized sources of human illness [12–14]. In line with increasing concern for non-O157 STEC, the US Department of Agriculture has amended food safety regulatory policy, declaring STEC of O26, O45, O103, O111, O121, and O145 serogroups as beef adulterants [15].

 Reports of hypervirulent clades or lineages of STEC O157 linked to human disease have been made. SNP analysis of 96 loci amongst 500 strains identified nine clades, including clade 8 linked with severe disease and designated hypervirulent [16]. Octamer-based genome scanning and length polymorphisms have identified three lineages (L) of O157; LI strains are represented in both cattle and human clinical isolates, and LII are predominantly from cattle [17–20]. Subsequent research examining Stx2 production showed differences across and within lineages. Sequencing of the *stx*_2_ flanking region demonstrates that LII has the transcriptional activator gene *Q* of L1 strains replaced by a *pphA* homologue. Further, LI and LI/II strains produced more Stx2 than LII, and LI strains of human origin produced more Stx2 than bovine LI [21].

 In British Columbia (BC), Canada, the rate of STEC infections have remained above the Canadian average since 2004, ranging between 2.4 and 4.3 cases/100,000 individuals [22]. However, no data exist describing the salient genetic features of STEC causing disease in BC or examined levels of antimicrobial resistance (AMR) in clinical isolates. To this end, we examined clinical STEC originating from BC using molecular and phenotypic methods to examine lineage, *stx* subtype, toxin production, presence of other virulence loci and plasmids, and AMR.

## 2. Materials and Methods

### 2.1. Strain Selection and Serotyping

 From the BCCDC Public Health Microbiology and Reference Laboratory Enteric Pathogen Monitoring Program during 2009-2010, we randomly selected 10 O157:H7 and 15 non-O157 STEC for genotypic and phenotypic characterization. All strains were propagated on Luria Bertani (LB) agar or broth (Becton Dickinson [BD], Mississauga, ON) and archived at −80°C in LB broth with 20% glycerol (Sigma Aldrich, Oakville, ON). Serotyping of all strains was performed using antisera from the Statens Serum Institute (Copenhagen, Denmark). 

### 2.2. Pulsed-Field Gel Electorphoresis

 Pulsed-field gel electrophoresis (PFGE) patterns were used to compare the genetic relatedness of isolates belonging to the same serogroup. Whole-cell isolation of DNA for PFGE analysis was prepared according to standard procedures [23]. Briefly, DNA-containing agarose plugs were subjected to endonuclease restriction using *Xba*I. Resulting fragments were separated using a CHEF DR III system (BioRad, Hercules, California). In each run, *Salmonella* Branderup standards were included every four lanes. PFGE pattern analysis was performed using Bionumerics v.6.0 software using standard comparison criteria [24]. 

### 2.3. Plasmid Analysis

 Plasmid was extracted (QIAprep Spin Miniprep; Qiagen, Toronto, ON) and 15 *μ*L-electrophoresed using Tris-acetate EDTA (TAE) buffer (70 V, 4-5 h). *Escherichia coli* EDL933 was used as a control. Plasmid size markers included the BAC-Tracker Supercoiled DNA Ladder (Epicentre, Markham, ON) and the Supercoiled DNA Ladder (Invitrogen, Burlington, ON).

### 2.4. Virulence Typing

 Genomic DNA was isolated using the DNeasy Blood and Tissue kit (Qiagen). Multiplex PCR was employed to detect the presence of *stx*_1_, *stx*_2_, *eaeA*, and *hlyA* [25]. All *stx* determinants were subtyped to identify *stx*_1_, *stx*_1c_, and *stx*_1d_ for *stx*_1_ and *stx*_2_, *stx*_2c_, *stx*_2d_, *stx*_2-O118_, *stx*_2e_, and *stx*_2g  _ for *stx*_2_ according to published methods [26, 27].

### 2.5. Comparative Genomic Fingerprinting (CGF)

 PCRs of 30 loci spanning the *E. coli* O157:H7 genome were used to fingerprint isolates as previously described [28]. Control strains included Sakai, ECI-272 and ECI-1717 for *E. coli* LI, I/II, and II, respectively, and K-12 (MG1655). Seven additional loci were used to increase genomic coverage and resolution (Table 1). For each locus, PCRs were repeated twice, with a positive result in either replicate being scored as presence (“1”) and a negative result in both replicates as absence (“0”).

PCR reactions were performed as previously described [28]. Amplicons were visualized on a QIAxcel using the QIAxcel DNA Screening Kit (Qiagen). Binary PCR data were analyzed by constructing an Euclidean distance matrix and hierarchically clustering strains using complete linkage. Analyses were performed in R (http://www.r-project.org/) using the heatmap.2 method of the gplots package (http://cran.r-project.org/web/packages/gplots/index.html). The image was colored using the GNU Image Manipulation Program v2.6.11.

### 2.6. Lineage Typing of STEC O157

 STEC O157:H7 EDL933 and Sakai and FRIK 2001 and ECI-1717 were used as LI and II controls, respectively. The LSPA was carried out according to Yang et al. [19] and analyzed as detailed by Sharma et al. [29].

### 2.7. AMR Profiling

 AMR phenotypes were determined by Kirby-Bauer disc diffusion assay. A 5 *μ*L volume of 18 h culture grown in Mueller Hinton broth (MH; BD) was mixed with 5 mL of molten agar (44°C) and overlaid on MH agar. Discs (BD) were placed on the agar surface and incubated (24 h, 37°C), and zones of inhibition measured to the nearest millimeter. Susceptibility was interpreted using CLSI guidelines [30]. In total, 19 antimicrobials were screened: amikacin (AMK; 30 *μ*g), amoxicillin/clavulanic acid (AMX; 30 *μ*g), ampicillin (AMP; 10 *μ*g), cefoxitin (FOX; 30 *μ*g), ceftazidime (CAZ; 30 *μ*g), ceftiofur (TIO; 30 *μ*g), chloramphenicol (CHL; 30 *μ*g), ciprofloxacin (CIP; 5 *μ*g), erythromycin (ERY; 15 *μ*g), gentamicin (GEN; 10 *μ*g), imipenem (IPM; 10 *μ*g), kanamycin (BCN; 30 *μ*g), nalidixic acid (NAL; 30 *μ*g), neomycin (NEO; 5 *μ*g), rifampicin (RIF; 5 *μ*g), spectinomycin (SPT; 100 *μ*g), streptomycin (STR; 10 *μ*g), tetracycline (TET; 30 *μ*g), and trimethoprim (TMP; 5 *μ*g). 

### 2.8. Genotypic Characterization of AMR

 Isolates were screened for the presence of class I, II, and III integrons [31, 32]. A multiplex PCR assay was used to detect the presence of CMY-2, CTX-M, OXA-1, SHV, and TEM *β*-lactamases [33]. DNA sequencing was used to confirm *bla*_CMY-2_ identity. 

### 2.9. AMR Plasmid Association

 Approximately 15 ng of plasmid was mixed with electrocompetent *E. coli* DH5*α* (Invitrogen). Following electroporation (MicroPulser, BioRad), cells were resuspended in SOC medium (Invitrogen), incubated (2 h, 37°C), plated on LB agar supplemented with AMP (100 *μ*g/mL), CHL (30 *μ*g/mL), or TET (30 *μ*g/mL), and incubated (24 h, 37°C). 

 Plasmid mobility in MDR strains was evaluated by conjugation with *E. coli* K802 (NAL^R^), *S. *Typhimurium MSC001 (NAL^R^), and *Citrobacter rodentium* MRS0026 (AMP^R^, NAL^R^). Due to intrinsic resistance to ampicillin, a nonpolar *bla* deletion was generated in *C. rodentium* MRS0026 (lambda-red system), rendering it AMP sensitive. Donors/recipients were grown for 18 h in LB broth (37°C) containing appropriate antimicrobials. For both, 400 *μ*L was centrifuged (2000 rpm, 10 min), washed, and resuspended in 400 *μ*L of fresh LB broth. Matings were incubated for 5 h at 37°C on a 400 *μ*L agar slant within a 1.5 mL microfuge tube. The mixture was resuspended in 100 *μ*L LB broth, plated on LB agar with antimicrobials, and incubated (24 h, 37°C). Transconjugants were streaked on LB agar and screened for AMR.

### 2.10. Quantification of Stx

 The production of Stx2 was quantified using polymixin lysis as described in Ziebell et al. [34], with some modification. Bacterial overnight cultures grown at 37°C with shaking (150 RPM) were diluted 1 : 250 and used to inoculate 5 mL of fresh brain heart infusion (BHI) broth in 50 mL Falcon tubes. Subsequently, 0.5 mg/mL of polymixin was added and incubated at 37°C for 1 h. Three experimental replicates were used to assess Stx2 toxin production. Total Stx was assessed using polymixin lysis [34], with slight modification. Cells were incubated with 0.5 mg/mL polymixin and incubated for 1 h at 37°C. Stx2 production differences between clusters were assessed using the *t*-test function of R.

## 3. Results

### 3.1. STEC Serotypes, Clonality, and Virulence Profiles

**Figure 1:**
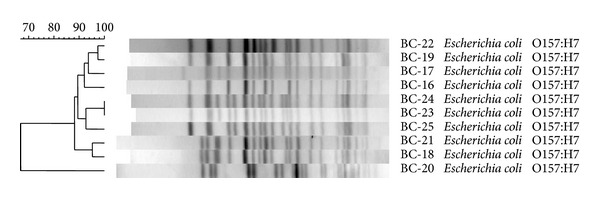
Clustering of STEC O157 by PFGE typing.

 In total, 10 serogroups and 12 unique serotypes were represented in the STEC panel. All O157 serogroup isolates displayed the H7 flagellar antigen. Non-O157 serogroups included O26, O121, and O165 NM variants; the remaining isolates were unique serotypes (Table 2). PFGE of STEC O157 isolates showed two indistinguishable isolates (BC-20 and -21), with all strains being distinguishable despite having ≥88% similarity (Figure 1). In spite of similar pulsotypes, BC-20 and -21 were distinguishable based on differing plasmid profiles and AMR phenotypes. Plasmid profiling revealed that 96% of isolates possessed plasmids which varied in size and number. Overall, 21 of 25 (84%) strains possessed a plasmid similar in size to pO157, with all STEC O157 possessing it. Two non-O157 STEC (O26:H11 and O111:NM) were shown to have seven plasmids.

 All *E. coli* O157:H7 were *eaeA* and *hlyA* positive whilst 13 and 12 of 15 non-O157 STEC were positive, respectively (Table 2). In non-O157 STEC, *stx*_1_ was observed more frequently than *stx*_2_, with 12 of 15 isolates encoding it and only six of 15 harboring *stx*_2_. Only three non-O157 STEC (O8:H16 and O165:NM) possessed both Shiga toxin genes. In contrast, eight of 10 STEC O157 had both, with all possessing *stx*_2_. Subtyping of *stx* revealed 19 of 20 STEC with Shiga toxin 1 subtyped as *stx*_1_; the remaining isolate (O146:H21) encoded *stx*_1c_. All STEC with Shiga toxin 2 had the *stx*_2_ subtype. LSPA typing showed STEC O157 strains were LI (LSPA-6 111111).

### 3.2. Comparative Genomic Fingerprinting

**Figure 2:**
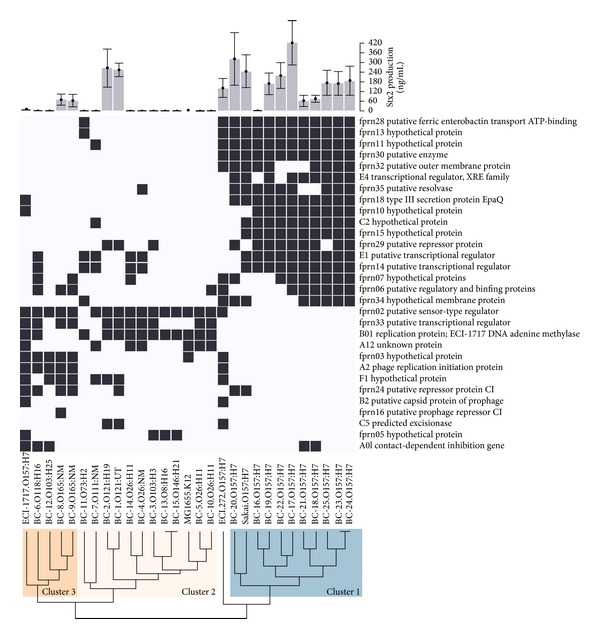
Hierarchical clustering and Stx2 production of 25 clinical STEC strains.

 All strains had unique CGF fingerprints, with the exception of two O157:H7 (BC-23 and BC-24) and two non-O157 (BC-13 and BC-15) isolates (Figure 2); the O157:H7 groupings mirrored those obtained by PFGE in that they generated identical strain clusters. The dendrogram in Figure 2 shows three clusters of STEC, with cluster (C) 1 consisting of O157:H7 LI strains, C2 of non-O157 STEC of serogroups O26, O146, O8, O121, O111, and O73, and the O103:H3 strain (BC-3), C3 including the O157:H7 LII control strain, all O165:NM and O118:H16 strains, and O103:H25 (BC-12), and C4 containing the O157:H7 LI/II control. C2 strains were positive for the fewest CGF loci, followed in an increasing order by C3, C4, and C1. 

### 3.3. Stx2 Production

 All O157:H7 strains produced Stx2, while only four of six non-O157 STEC encoding *stx*_2_ did, including O121 (BC-1, BC-2) from C2 and O165:NM (BC-8, BC-9) from C3 (Figure 2). Both O121 strains produced levels of Stx2 similar to the STEC O157 Sakai strain. Of the strains that produced Stx2, C1 and C2 strains produced significantly more than C3 strains (*P* = 0.018 and *P* = 0.005, respectively). There was no significant difference in Stx2 production between C1 and C2 strains (*P* = 0.072).

### 3.4. AMR

 All isolates were sensitive to AMK, CIP, GEN, IMP, NAL, RIF, and TMP (Tables 2 and 3). However, 11 of 25 were resistant to at least one antibiotic while reduced susceptibility (RSC) was observed in all remaining strains, particularly to NEO (*n* = 16), SPT (*n* = 12), TET (*n* = 5), BCN (*n* = 3), and STR (*n* = 3). The most common resistance was to NEO (*n* = 6), STR (*n* = 5) and TET (*n* = 4). CHL resistance and resistance/RSC to BCN were infrequently observed. Three O157 and two non-O157 STEC were resistant to ≥3 antibiotics, though only O165:NM (BC-8) possessed resistance/RSC to three different classes. Six multidrug resistant (MDR) profiles were observed, including NEO-STR, NEO-SPT, and BCN-NEO-TET in non-O157 STEC (Table 4). These were observed less frequently (0 to 40%) in the 10 O157:H7 isolates. Whilst four of 10 O157:H7 STEC were resistant/RSC to one antibiotic, only three of 15 non-O157 were singularly resistant. Interestingly, BC-20 possessed resistance to AMP, CAZ, and TIO, suggesting the presence of a beta-lactamase affording resistance to extended spectrum cephalosporins (ESC). 

### 3.5. Molecular AMR Characterization and Mobility

 No integrons were detected in any isolate. In BC-20, the presence of *bla*_CMY-2_ was confirmed by PCR and DNA sequencing. Transformants were shown to possess a similar 70 kb plasmid and resistance profile and were positive for *bla*_CMY-2_. Mating experiments showed that resistance was transferable to *E. coli*, *S. *Typhimurium, and *C. rodentium* through conjugation. Transformations and conjugations were performed using other MDR STEC (Table 5). With the exception of *E. coli* O118:H16 (BC-6), all strains readily transferred resistance.

## 4. Discussion

Boerlin et al. [35] reported an association between clinical EHEC serotypes and *stx*_2_ and *eae* and, to a lesser extent, *hlyA*. More recently, it was reported that lineage and isolation origin correlate with Stx2 production [21]. Specifically, human LI isolates produce more toxin than cattle LI and LII strains. Also, high-toxin producing LI strains encode *stx*_2_ whereas LII strains possess *stx*_2c_, and LI/II have both. In this study, all *E. coli* O157:H7 isolates belonged to LI (LSPA-6 111111) and carried the *stx*_2_ subtype. This is consistent with observations made by Sharma et al. [29] who reported 91.6% of clinical strains in Alberta typed as LSPA-6 111111 and elsewhere [17–19]. However, it was recently shown by Franz et al. [36] and Mellor et al. [37] that the majority of clinical STEC O157 in The Netherlands, Argentina, and Australia, respectively, are LI/II strains. As such our study provides further evidence demonstrating that disease-causing STEC O157 in North America differ from STEC causing disease on other continents. When Shiga toxin production was examined, while levels of Stx2 associated with O157 strains were variable, these strains clustered together by CGH and generally produced more Stx2 than non-O157 STEC strains possessing *stx*_2_. Interestingly, STEC O157 BC-17 produced higher levels of toxin than the Sakai strain.

 Virulence profiles in non-O157 isolates displayed more variability than O157 STEC. Buvens and Piérard [38] reported a progressive decrease of O-island (OI) 122 components (*nleB*, *nleE*) when examining seropathotypes A to D. Further, OI-122 and the presence of *stx*_2_, *eae*, and *espP* were present at higher rates in non-O157 causing HUS. Although we did not screen for the presence of OI-122, only five non-O157 STEC possessed both *stx*_2_ and *eae*. Interestingly, while three of these isolates lacked *hlyA*, four were the only non-O157 STEC to show significant Stx2 production. 

 This CGF scheme was developed for O157:H7 STEC [28]. Overall, it performed well, though in one case two STEC from unrelated serogroups (BC-9 and BC-15) were indistinguishable, possessing only three of 30 loci. Previous studies reported low frequencies of O157:H7-specific elements in non-O157 STEC, suggesting independent acquisition of non-O157:H7 traits and that these traits not be included in our CGF scheme [39–41]. Generally, non-O157:H7 strains sharing more elements with O157:H7 STEC grouped into seropathotypes associated with more severe human disease. 

 Seropathotyping [6] has been useful in assigning risk but is broad in scope. Here O26:H11 (BC-14) was found to differ at 10% of CGF loci from the other O26:H11 strains (BC-5, BC-10). Thus, similar to O157:H7, non-O157 STEC may contain distinct lineages differing in genomic content and their capacity to cause disease. While the resolution provided by seropathotyping and the O157 CGF are helpful in differentiating among strains, complete genome sequence analysis of non-O157 STEC will be required to identify lineage-specific loci among them and the existence of unique “genopathotypes.” 

 Previous research found only LII O157:H7 STEC possessed *cdi* [42], which is common in uropathogenic *E. coli* [43]. In this collection, we observed two LI strains to be positive for *cdi*. This implies that these isolates have either recently acquired this gene or the original study was not sufficiently broad to capture LI diversity.

 At this time, antibiotic administration for human STEC infection is contraindicated in the treatment of STEC infections in North America [44], though conflicting reports of clinical outcomes and antibiotic administration have been made. Antibiotic usage for treatment has been linked to diminished clinical outcomes [45, 46] or had little influence on patient outcomes [47, 48]. In contrast, early administration of fosfomycin was reported to improve clinical outcomes [49]. Recent data from the *E. coli* O104:H4 outbreak support administration of antibiotics, with fewer seizures, deaths, and surgeries required for antibiotic-treated patients [50]. Further, treated individuals experienced shorter symptom duration and shed the pathogen for significantly less time, thus posing a lower risk of secondary disease transmission [51]. For these reasons, AMR data observed in clinical STEC strains is needed to provide appropriate therapeutic guidance to physicians should the current contraindication be rescinded.

 With the recognition of STEC as a significant source of human disease, increased reports of AMR have been made in recent years [14, 52–58]. In this study, though clinical STEC were sensitive to many drugs, RSC in all examined strains examined or resistance in 11 of 25 strains to at least one antibiotic was observed. Similar sensitivity to AMK, various *β*-lactams, CIP, and TMP has been reported in STEC of diverse origin [53, 54, 56]. Although the sample size in our study is small, levels of AMR were high considering the clinical origins of the strains. For example, in the US higher AMR levels were reported in cattle (34%) and food (20%) compared to clinical (10%) isolates [59]. Similarly, in Spain STEC O157 resistant to one or more antimicrobials were recovered in 53% of bovine and 57% of beef isolates, but only 23% of clinical isolates were resistant [52]. In Alberta, Canada, whilst 34% of bovine isolates were resistant to one of more antimicrobials, only 10% of clinical isolates were resistant with the most commonly observed resistances being to STR, sulfisoxazole, and TET [29]. Although resistance to sulfisoxazole was not screened in our study, NEO, STR, and TET were the most frequently occurring AMR phenotypes. 

Notably, BC-20 possessed a plasmidic *bla*_CMY-2_. Considering the highly promiscuous nature of plasmid harbouring observed *bla*_CMY-2_ in this study and previously reported plasmids encoding it [60, 61], it is not surprising that generic *E. coli* and STEC O157 possessing a similar plasmid have been recovered from cattle hides, carcasses, processing environments, and ground beef in Canada [62, 63]. Our observation of a clinical STEC possessing RSC to critically important therapeutic agents suggests caution in the administration of ceftriaxone and other therapeutic ESCs that may be considered for treatment of STEC infections.

## 5. Conclusions

 We observed a small collection of clinical STEC in BC to be of variable genomic content. STEC O157 were LI strains producing significant amounts of Stx2. Based on CGF, increased genomic variation was observed in non-O157 STEC strains, with isolates clustering into two distinct groups. CGF and Stx2 assays suggest that serogroups O121 and O165 were more similar to STEC O157 in genetic content than other non-O157 and that these isolates may produce high levels of Stx. However, further work examining clinical O121 and O165 serogroup strains is required to substantiate this assertion. Although this may make these serogroups of greater public health concern than other non-O157 STEC, surveillance data is required to examine the frequency and disease severity that these serogroups cause in human STEC infections. Despite the clinical origins of the non-O157 strains, the genetic variability revealed by our CGF strategy highlights the need for more detailed genetic information, such as that offered by whole genome sequencing. Lastly, we also observed high levels of AMR and RSC in this clinical collection, including a highly mobile *bla*_CMY-2_-encoding plasmid conferring resistance to clinically relevant treatment options. Considering recent evidence suggesting that antimicrobial therapy may lead to reduced severity of clinical outcomes, further data examining AMR in STEC seem prudent. 

## Figures and Tables

**Table 1:** The seven additional comparative genomic fingerprinting primers used in this study, their genomic location, and function of target region. The annealing temperature used for all primers was 55°C.

Primer name	Forward sequence (5′-3′)	Reverse sequence (5′-3′)	O157:H7 strain: genome location (bp)	Function
A2	ACGGTTTCGCGCAGCTCCTCTT	GCCTGATGCGCACGGCATTCAA	EC4115: 2834437…2834633	Phage replication initiation protein
B2	GGTGCTCAAGCAGCGCCACAAA	TGCCGTTGCTTTGCCTGCCATT	EC4115: 1542851…1543021	Putative capsid protein of prophage
C5	TGGGAGGGTGCATGTAAGGCGT	TGGGGCATGAACTTGGGGGAGT	EC4115: 3295343…3295780	Predicted excisionase
F1	TCGCAGGTATGGGTGCTGCTGT	ACGACGAAGCTTACCCTGCTGC	EC4115: 5180205…5180308	Hypothetical protein
E4	TGCAAAGGCATGGGTCCCAACG	TGATGCGGCAGCTATGGTCGCA	Sakai: 2933058…2933350	Transcriptional regulator, XRE family
E1	AGTTCGCCAGTAGGCTTGCGCT	TTCGACGGGCAATTTCTGCCTGC	Sakai: 1929526…1929606	Putative transcriptional regulator
C2	AGGCATGCGACCTTTCTAACTGGCA	TCTTCAGCGGCTGCCTGATATGCT	Sakai: 1936190…1936337	Hypothetical protein

**Table 2:** Antimicrobial resistance, serotypes, PFGE, plasmid, and virulence profiles of clinical STEC isolated from British Columbia.

Strain no.	Serotype	Virulence genes				*Xba*I PFGE profile	Plasmid profile (kb)	AMR phenotype
		*st* *x*_1_^a^ (subtype)	*st* *x*_2_ (subtype)	*ea* *eA*	*hl* *yA*			
BC-13	O8:H16	+	+ (*stx*_2_)	−	+	ECXA1.2261	100, 16, 12, 8, 7	BCN^I^, NEO^I^, SPT^I^, STR^I^
BC-14	O26:H11	+	−	+	+	ECXA1.2513	93, 14, 7	NEO, TET^I^
BC-5	O26:H11	+	−	+	+	ECXA1.2515	93, 80, 14, 7, 6, 3.5, 2.5	NEO^I^
BC-10	O26:H11	+	−	+	+	ECXA1.2280	93, 14, 7	NEO^I^, SPT^I^
BC-4	O26:NM	+	−	+	+	ECXA1.2516	93, 14, 7	NEO^I^, SPT^I^
BC-11	O73:H2	−	+ (*stx*_2_)	+	+	ND^b^	100	BCN^I^, SPT^I^, STR, TET^I^, NEO^I^
BC-3	O103:H3	+	−	+	+	ECXA1.2517	100, 93, 14, 7, 5	BCN, NEO, SPT^I^, TET
BC-12	O103:H25	+	−	+	+	ECXA1.2262	93	STR, NEO^I^
BC-7	O111:NM	+	−	+	+	ND	93, 80, 65, 14, 7, 6, 3.5	NEO^I^
BC-6	O118:H16	+	−	+	+	ND	93, 14, 7, 6	BCN^I^, KAN^I^, NEO, SPT, STR, TET
BC-2	O121:H19	−	+ (*stx*_2_)	+	−	ECXA1.2518	None	NEO^I^, SPT^I^
BC-1	O121:UT	−	+ (*stx*_2_)	+	+	ECXA1.2518	93	NEO, SPT^I^
BC-15	O146:H21	+ (*stx*_1c_)	−	−	+	ND	80, 15, 12, 8	NEO^I^
BC-8	O165:NM	+	+ (*stx*_2_)	+	−	ECXA1.2514	93	AMP^I^, NEO^I^, SPT, TET^I^
BC-9	O165:NM	+	+ (*stx*_2_)	+	−	ECXA1.2514	93	NEO^I^, TET^I^
BC-16	O157:H7	−	+ (*stx*_2_)	+	+	ECXA1.0023	93	NEO
BC-17	O157:H7	+	+ (*stx*_2_)	+	+	ECXA1.2426	93	SPT^I^
BC-18	O157:H7	+	+ (*stx*_2_)	+	+	ECXA1.2203	93, 80, 65	CHL, NEO^I^, SPT^I^, STR, TET
BC-19	O157:H7	+	+ (*stx*_2_)	+	+	ECXA1.0001	93, 70	TET^I^
BC-20	O157:H7	+	+ (*stx*_2_)	+	+	ECXA1.2412	93, 70, 3.5	AMC, AMP, FOX, CAZ, TIO, STR^I^
BC-21	O157:H7	+	+ (*stx*_2_)	+	+	ECXA1.2412	93, 80, 65	CHL, NEO^I^, STR, TET
BC-22	O157:H7	−	+ (*stx*_2_)	+	+	ECXA1.2203	93, 80, 50, 30	NEO^I^
BC-23	O157:H7	+	+ (*stx*_2_)	+	+	ECXA1.0854	93	NEO^I^, SPT^I^
BC-24	O157:H7	+	+ (*stx*_2_)	+	+	ECXA1.1107	93	NEO, SPT^I^
BC-25	O157:H7	+	+ (*stx*_2_)	+	+	ECXA1.2397	93	NEO^I^, SPT^I^

^a^All were subtype *stx*_1_ with a single exception; ^b^not determined; ^ I^denotes reduced susceptibility.

**Table 3:** Antimicrobial resistance (AMR) amongst Shiga toxin-producing *E. coli*.

Antimicrobial agents	STEC AMR susceptibility (%)			% AMR	
	Susceptible	Reduced susceptibility	Resistant	Non-O157 STEC (*n* = 15)	O157 STEC (*n* = 10)
Aminoglycosides					
Amikacin	100	0	0	0	0
Gentamicin	100	0	0	0	0
Kanamycin	84	12	4	7	0
Neomycin	0	76	24	27	20
Streptomycin	72	8	20	20	20
Penicillin					
Ampicillin	92	4	4	0	10
Carbapenem					
Imipenem	100	0	0	0	0
Cephalosporin					
Ceftazidime	96	0	4	0	10
Macrolide					
Erythromycin	0	0	100	100	100
Quinolones					
Ciprofloxacin	100	0	0	0	0
Nalidixic acid	100	0	0	0	0
Phenicol					
Chloramphenicol	92	0	8	0	20
Ansamycin					
Rifampicin	100	0	0	100	100
Spectinomycin					
Spectinomycin	44	48	8	13	0
Tetracylines					
Tetracycline	64	20	16	13	20
Sulfonamide					
Trimethoprim	100	0	0	0	0

**Table 4:** Multidrug resistant and reduced susceptibility STEC phenotype patterns.

Common antibiogram profiles	AMR	
	No. non-O157 STEC (%)	No. O157 STEC (%)
NEO, STR	10 (67)	2 (20)
NEO, SPT	9 (60)	4 (40)
BCN, NEO, TET	3 (20)	0
SPT, STR, TET	2 (13)	0
CHL, STR, TET	0	2 (20)
AMP, CAZ, AMC, FOX, CFT	0	1 (10)

**Table 5:** AMR phenotype of transformants and transconjugants derived from STEC plasmid DNA and matings, respectively.

Serotype (strain ID)	Transferred AMR phenotype	Transformants	Transconjugants			
		*E. coli* DH5*α*	*E. coli* K802NR	*C. rodentium* DBS100	*C. rodentium* MCS026^a^	*S.* Typhimurium MCS001
O103:H3 (C)	BCN, NEO, TET	+	+	+	n/a^b^	+
O118:H16 (F)	SPT, STR, TET	−	−	−	n/a	−
O157:H7 (3)	CHL, STR, TET	+	+	−	n/a	n/a
O157:H7 (5)	AMP, CAZ, AMC, FOX, TIO	+^c^	+^c^	n/a	+	+
O157:H7 (6)	CHL, STR, TET	+	+	−	n/a	n/a

^a^ *C. rodentium* MCS026 was constructed by deleting *bla* in *C. rodentium* DBS100.

^b^Not applicable.

^c^Reduced susceptibility.
